# The cytotoxic mechanisms of disulfiram and copper(ii) in cancer cells

**DOI:** 10.1039/c5tx00210a

**Published:** 2015-09-10

**Authors:** Patricia Erebi Tawari, Zhipeng Wang, Mohammad Najlah, Chi Wai Tsang, Vinodh Kannappan, Peng Liu, Christopher McConville, Bin He, Angel L. Armesilla, Weiguang Wang

**Affiliations:** a Research Institute in Healthcare Science , Faculty of Science & Engineering , University of Wolverhampton , Wolverhampton WV1 1LY , UK . Email: w.wang2@wlv.ac.uk ; Fax: +44 (0)1902 322714 ; Tel: +44 (0)1902 322756; b Medicine & Healthcare Science , Faculty of Medical Science , Anglia Ruskin University , UK; c School of Chemistry , University of Birmingham , UK; d National Engineering Res Centre for Biomaterials , Sichuan University , China; e School of Pharmacy , Faculty of Science & Engineering , University of Wolverhampton , Wolverhampton WV1 1LY , UK

## Abstract

The anticancer activity of disulfiram (DS) is copper(ii) (Cu)-dependent. This study investigated the anticancer mechanisms of DS/Cu using *in vitro* cytotoxicity and metabolic kinetic analysis. Our study indicates that DS/Cu targets cancer cells by the combination of two types of actions: (1) instant killing executed by DS/Cu reaction generated reactive oxygen species; (2) delayed cytotoxicity introduced by the end product, DDC-Cu. Nanoencapsulation of DS might shed light on repositioning of DS into cancer treatment.

## Abstract 2

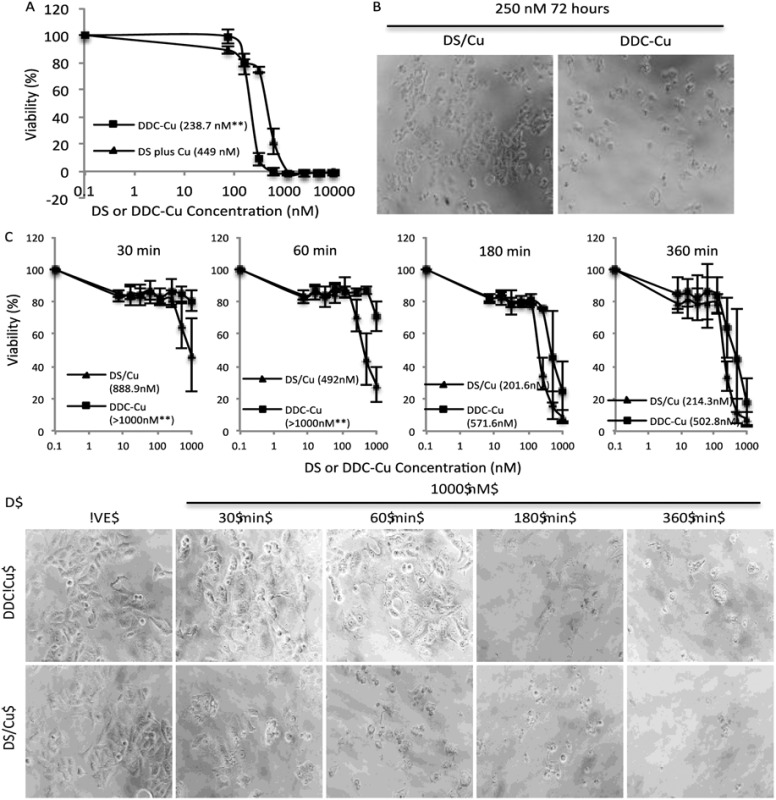
The anticancer activity of disulfiram (DS) is copper(ii) (Cu)-dependent.

## Introduction

Disulfiram (DS), an anti-alcoholism drug used in clinic for over 60 years,^1^ demonstrates excellent *in vitro* anticancer activity in a wide range of cancer cell lines.^2–11^ The potential molecular anticancer mechanisms of DS include inhibition of proteasome/NFκB pathway,^3,12^ MDR1,^13^ topoisomerase and MMP.^14^ DS is also an irreversible aldehyde dehydrogenase (ALDH) inhibitor targeting cancer stem cells.^7–9,11^ The *in vitro* cytotoxicity of DS is entirely dependent on supplement of copper(ii) (Cu) or some other transition bivalent metal ions in the culture medium.^2,6,7,15–17^ Cu plays a crucial role in redox reactions and triggers generation of reactive oxygen species (ROS), which damage DNA, protein and lipids leading cells into apoptosis. Although the concept of using Cu to tackle cancer was proposed many decades ago,^18^ it has never demonstrated clinical anticancer efficacy. This is partially due to the strict control of Cu transport into cancer cells by the *trans*-membrane Cu transporter Ctr1.^3^ Diethyldithiocarbomate (DDC), the derivative of DS, chelates copper to form a DDC-Cu complex and transports Cu into cancer cells.^2,11^ Supplementing with Cu, DS is highly toxic to cancer cells *in  vitro*.^2,6,7,15–17^

Although prompted by the very promising lab data, several clinical trials using oral version of DS plus copper gluconate in cancer treatment have been completed or on-going (https://clinicaltrials.gov/ct2/results?term=disulfiram+AND+cancer&Search=Search), no positive data have been published. Therefore, elucidating the discord between the anticancer activity of DS in laboratory and clinic is of significant clinical importance. Recently it was suggested that the *in vitro* cytotoxicity of DS in cancer cells is introduced by ROS generated from the reaction of DDC and Cu rather than the final product, DDC-Cu. The detail of the ROS generated from DDC and Cu reaction is presented in the Scheme 2 of Lewis’ recent publication.^19^ DS and Cu may not be able to react near the cancer cells *in vivo*. Considering the extremely short half-life of ROS,^20^ it may be impossible to translate the *in vitro* cytotoxic effect of DS into clinic. The present study intends to answer a very serious challenge: if DS can be repositioned into cancer indication?

## Results and discussion

**Fig. 1:**
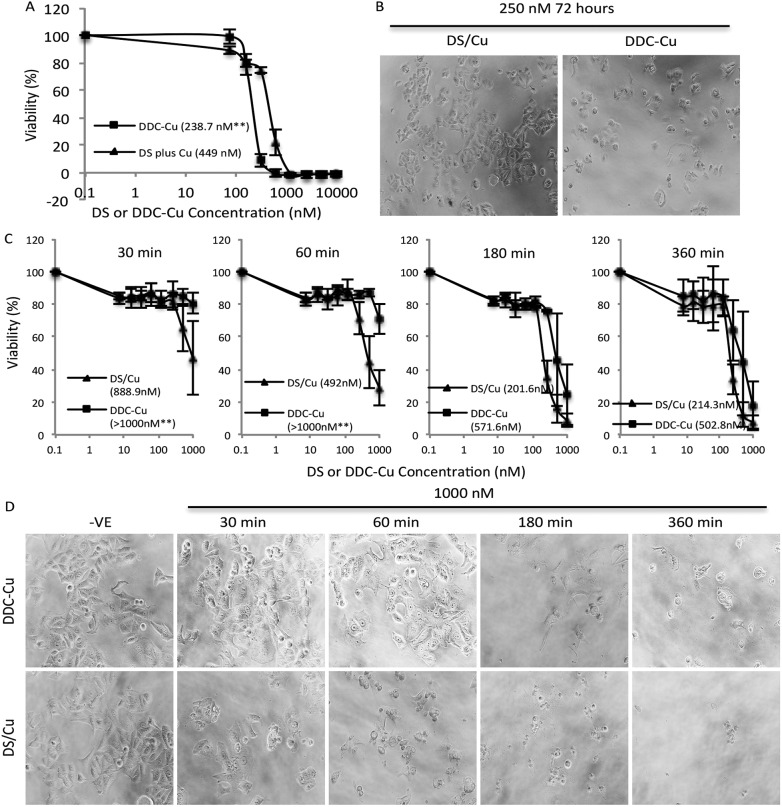
Cytotoxicity of DS/Cu and DDC-Cu on MCF7 cells. A and B. After 72 h exposure, DDC-Cu showed higher cytotoxicity to cancer cells. C and D. In comparison with DDC-Cu, DS/Cu demonstrated earlier killing effect. Inserted figures in A and C: IC_50s_; **p < 0.01.

Firstly, we set up an *in vitro* assay to compare the cytotoxicity of DDC-Cu and DS plus Cu (DS/Cu). If the cytotoxicity of DS in cancer cell is only introduced by the DS/Cu reaction, the DDC-Cu should not show significant cytotoxicity. The MCF7 breast cancer cells (5 × 10^3^ per well) were cultured in 96-well plates and then subjected to the treatment of DS/Cu [equal molar ratio of DS (Sigma, Dorset, UK; in DMSO) and CuCl_2_ (in H_2_O)] or DDC-Cu (TCI, Merseyside, UK; in DMSO). After 72 hours exposure, the cells were subjected to a typical *in vitro* MTT assay.^21^ In contrast to our original hypothesis, both DDC-Cu and DS/Cu are highly cytotoxic to MCF7 cells (Fig. 1A and B). In comparison with DS/Cu (IC_50_: 449 nM), DDC-Cu is even more cytotoxic (IC_50_: 238.7 nM, *p* < 0.01). Our finding indicates that the cytotoxicity of DS/Cu is not solely caused by the reaction of DS and Cu. DDC-Cu, the final product of the reaction, may play more important role in DS/Cu induced cell death.

To determine the time-dependent cytotoxicity of DS/Cu reaction and DDC-Cu, we performed the following experiments. The cancer cells were plated on 96-well plates and exposed to DDC-Cu or DS/CuCl_2_ (1 : 1 molar ratio). After 30, 60, 180 and 360 min drug exposure, the cells were cultured for another 72 hours in drug-free medium and subjected to MTT assay. The massive cell death was observed after 30 min exposure to DS/Cu and further killing was detected until 180 min exposure (Fig. 1C and D). This is highly in line with the UV-Vis absorption time-course plot of DS/Cu reaction previously published.^19^ No significant cell killing was observed within 30 min exposure to DDC-Cu but markedly intensified after 180 min exposure (Fig. 1C and D).

**Fig. 2:**
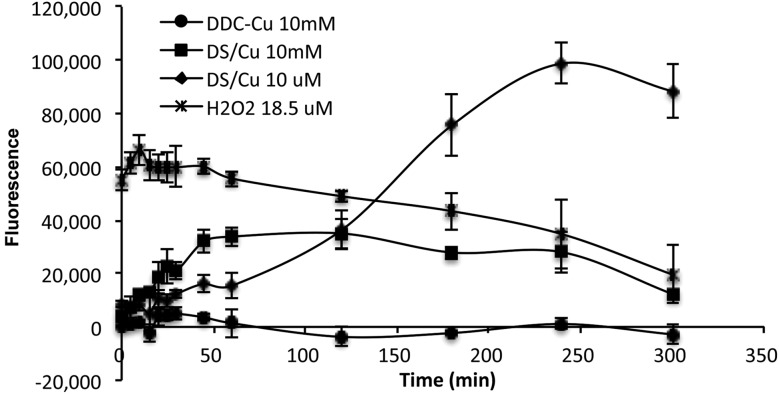
The ROS activity detected in DS/Cu, DDC-Cu and H_2_O_2_ containing cell culture medium. The strength of fluorescence represents the intensity of ROS in the medium.

ROS is a group of reactive oxygen-containing chemical species, including the superoxide anion (O_2_^–^), hydrogen peroxide (H_2_O_2_) and the hydroxyl radical (HO˙), which is biologically generated from the mitochondrial respiratory chain reaction in living cells. The half-life of ROS is only 10^–9^ s (HO˙) to 1 ms (H_2_O_2_).^20^ When equal molar ratio of DS and Cu are mixed, the reaction is instantly triggered and completed within 150 min.^19^ Therefore, if the cytotoxicity is induced by the extracellular ROS generated from DS/Cu reaction, the cytotoxic effect should be observed instantly after drug exposure. Further more, we examine the ROS generation from DS/Cu reaction (Fig. 2). Equal molar concentration (10 μM or 10 mM) of DS and CuCl_2_ was mixed in culture medium. The ROS generated in the system was detected by OxyBURST H2HFF Green Assay (Invitrogen, Paisley, UK) following the supplier's instruction. High levels of ROS were detected in DS/Cu medium. At working concentration (10 μM), DS/Cu generated significantly higher ROS than those from 18.5 μM H_2_O_2_ and was maximised within 4 hours. Lower levels of ROS were detected in 10 mM DS/Cu reaction which may be due to the instant crystallization of DS and Cu at higher concentration. No ROS was detected in the DDC-Cu containing medium.

Furthermore, we examined the metabolic kinetics of DS/Cu and DDC-Cu in cell culture. MCF7 cells were treated with equal molar ratio of DS and CuCl_2_ at a final concentration of 2 μM. The medium and cells were separately collected after 30, 180 and 360 min. The reaction products of DS/Cu in the whole cell lysate and medium were extracted in 0.25 ml chloroform and subjected to HPLC analysis. The separation was achieved using a C18 reverse-phase column at an injection volume of 20 μl and a flow rate of 1 ml min^–1^. The wave length of 435 nm and 275 nm, mobile phase of 10 : 90% and 30 : 70% (water : methanol, v/v) were used for DDC-Cu and DS analysis respectively.

**Fig. 3:**
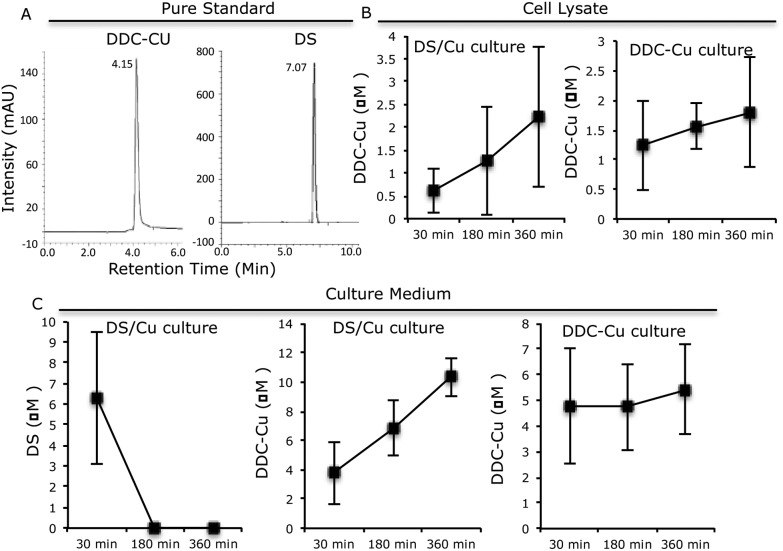
The kinetic of DS and DDC-Cu in the cell culture. A. The HPLC profiles of DS and DDC-Cu. B. The time-dependent trend of DDC-Cu in the cell lysate. C. The time-dependent trend of DS and DDC-Cu in culture medium. *N* = 3, Bar: SD.

The pure standard DDC-Cu and DS were detected at retention time of 4.15 and 7.07 min respectively (Fig. 3A). The DDC-Cu was detected in both medium and cell lysate extracts after culturing in DS/Cu-containing medium for 30 min. The peak was steadily increased in the medium and cell lysate over 360 min (Fig. 3B and C). The stable levels of DDC-Cu were detected in the medium and cell lysate when pure DDC-Cu compound was added into the cell culture (Fig. 3B and C). DS was detected in the DS/Cu cultured medium at 30 min and sharply dropped to undetectable level (Fig. 3C). No DS was detected in the cell lysate. These results indicate that DS/Cu reaction generated DDC-Cu can penetrate into and cumulate within cancer cells (Fig. 3B) to induce apoptosis.

**Scheme 1:**
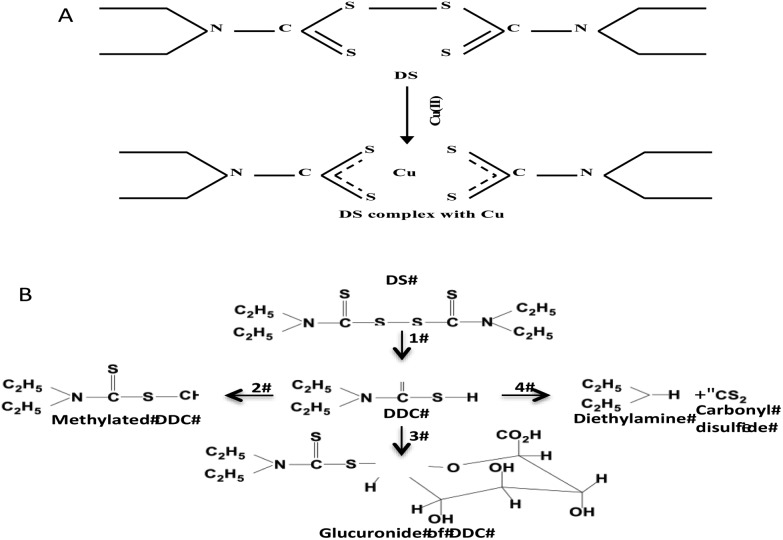
A. Chelation of DS and Cu. B. The DS metabolism pathways. 1. DS is rapidly reduced to DDC by Glutathione reductase; 2. DDC is promptly converted to methylated DDC by S-methylation; 3. DDC is conjugated with glucuronide; 4. DDC is also non-enzymatically degraded into diethylamine and carbonyl desulfid.

The findings from this study suggest that the cytotoxicity of DS/Cu may be introduced from the following two actions. (1) DS/Cu reaction-generated ROS: This is an instant and short-term action; (2) toxic effect of DDC-Cu: DDC-Cu is an end product with delayed but stronger long-lasting effect. The chelation of DS with Cu is indispensible for both of these two actions. Scheme 1 shows the metabolic pathways of DS. *In vivo*, DS is instantly reduced to DDC in the bloodstream, which is also very unstable and promptly converted into the irreversible downstream metabolites, *e.g.* DDC-glucuronide, methylated DDC and other degraded products. In all of these products, the functional sulfhydryl group of DDC is destroyed making DDC lose its chelating ability. Our unpublished data show that DS is undetectable after mixed with horse serum for 2 min. Due to the very short half-life of DS and DDC in the bloodstream, no ROS and DDC-Cu will be generated if the oral version of DS and Cu is administered to patient separately. This may explain the discrepancy between *in vitro* experiment and clinic. To resolve this problem, we recently protected DS from degradation in the bloodstream by encapsulation of DS into nanoparticles. This strategy significantly extended the half-life of DS. We have demonstrated that in combination with oral administration of copper gluconate, intravenous version of nanoencapsulated DS showed significantly stronger anticancer efficacy in mouse breast, lung and brain cancer xenograft models (ref. 9 and unpublished data). Therefore, nanomedicine may be a novel strategy for translation of DS into cancer indication. In contrast to DS, DDC-Cu is a very stable chemical with a half-life in serum for more than 4 hours (our unpublished data). It can potentially be another druggable candidate for cancer therapeutics.

## Conclusion

Our results demonstrate that DS/Cu induces cell death *via* instant and delayed phases. The instant phase may be solely caused by the DS/Cu reaction generated ROS. The delayed phase may follow the rule of conventional anticancer drugs, which may interfere the vital molecular pathways within the cancer cells and induce apoptosis. Although the reaction-induced cytotoxicity was started earlier, DDC-Cu showed significantly stronger anticancer activity after 2 to 3 doubling time.

We acknowledge support from the Tertiary Education Trust Fund Niger Delta University, Nigeria for PET's PhD studentship and Marie-Curie IIF Program (PIIF-GA-2013-629478) for ZPW.
